# Supplementary material for: Maternal immune response and placental antibody transfer after COVID-19 vaccination across trimester and platforms
Source: Nat Commun. 2022 Jun 28;13:3571. doi: 10.1038/s41467-022-31169-8 (PMC9239994; doi:10.1038/s41467-022-31169-8)
Supplement: Supplementary file 1 — Supplemental Information [file 41467_2022_31169_MOESM1_ESM.docx]

Supplemental Information

Supplemental Table 1. Pregnancy characteristics of systems serology maternal:neonatal dyads, by vaccine platform.

|  | Overall  (N=123) | Ad26.COV2.S  (N=21) | mRNA-1273  (N=47) | BNT162b2  (N=55) | *P* |
| --- | --- | --- | --- | --- | --- |
| Gestational age at delivery, completed weeks | 39 [38, 40] | 39 [39, 40] | 39 [38, 40] | 39 [39, 40] | 0.57 |
| Mode of delivery (%) | | | | | |
| Vaginal delivery | 88 (72) | 13 (62) | 36 (77) | 39 (71) | 0.45 |
| Cesarean section | 35 (28) | 8 (38) | 11 (23) | 16 (29) |  |
| Neonatal sex (%) | | | | | |
| Male | 73 (59) | 11 (52) | 28 (60) | 34 (62) | 0.76 |
| Female | 50 (41) | 10 (48) | 19 (40) | 21 (38) |  |
| Neonatal birthweight, g | 3362 [3094, 3741] | 3390 [3155, 3820] | 3345 [3024, 3730] | 3395 [3141, 3716] | 0.90 |
| Prior infection with SARS-CoV-2* (%) | | | | | |
| No | 119 (97) | 19 (90) | 45 (96) | 55 (100) | 0.06 |
| Yes | 4 (3) | 2 (10) | 2 (4) | 0 (0) |  |
| Trimester of vaccination (%) | | | | | |
| First | 3 (2) | 0 (0) | 3 (6) | 0 (0) | <0.001 |
| Second | 70 (57) | 9 (43) | 36 (77) | 25 (45) |  |
| Third | 50 (41) | 12 (57) | 8 (17) | 30 (55) |  |
| Time from vaccination to delivery†, days | 74 [38, 100] | 75 [52, 95] | 82 [61, 108] | 50 [20, 78] | 0.002 |

*Three participants tested positive for SARS-CoV-2 prior to vaccination and one participant tested positive at delivery †Defined time from single dose of Ad26.COV2.S vaccine or second dose of mRNA-1273 or BNT162b2 and sample collection at delivery. Continuous variables presented as median [IQR] and categorical variables as n (%). Differences between groups assessed by Kruskal-Wallis (continuous) or Fisher’s exact test (categorical).

Supplemental Table 2. Characteristics of maternal:neonatal dyad cohort for transplacental antibody transfer ELISA analysis by trimester of vaccination.

|  | All  (N=175) | First  (N=39) | Second  (N=79) | Third  (N=57) | *P* |
| --- | --- | --- | --- | --- | --- |
| Vaccine type (%) | | | | | |
| Ad26.COV2.S | 27 (15) | 3 (8) | 12 (15) | 12 (21) | <0.001 |
| mRNA-1273 | 62 (35) | 14 (36) | 40 (51) | 8 (14) |  |
| BNT162b2 | 86 (49) | 22 (56) | 27 (34) | 37 (65) |  |
| Prior infection with SARS-CoV-2* (%) | | | | | |
| No | 164 (94) | 34 (87) | 75 (95) | 55 (96) | 0.22 |
| Yes | 11 (6) | 5 (13) | 4 (5) | 2 (4) |  |
| Time from vaccination to delivery†, days | 82 [45, 127] | 167 [156, 177] | 92 [74, 109] | 28 [19, 43] | <0.001 |

*Three participants tested positive for SARS-CoV-2 prior to vaccination and one participant tested positive at delivery †Defined time from single dose of Ad26.COV2.S vaccine or second dose of mRNA-1273 or BNT162b2 and sample collection at delivery. Continuous variables presented as median [IQR] and categorical variables as n (%). Differences between groups assessed by Kruskal-Wallis (continuous) or Fisher’s exact test (categorical).

**Supplemental Figures**

Figure S1. Vaccine platform results in different vaccine-induced antibody induction in maternal samples

1. Spike-specific IgG2, IgG3, IgM and IgA were measured by Luminex. The dot plots show the titer for mothers who received Ad26.COV2.S (red), mRNA-1273 (yellow) or BNT162b2 (blue).
2. The heatmaps show the spearman correlation of antibody features against the Spike of the ancestral virus versus antibody features against the Spikes from variants of concern in cord blood, analyzed separately for each vaccine platform. Orange indicates a positive spearman correlation, * p < 0.05, ** p < 0.01, *** p < 0.001, **** p < 0.0001
3. The dot plots show the Spike-specific antibody-dependent NK cell activation, as measured by % MIP-1b + and % IFN-g+ NK cells, in maternal blood. Significance was determined by Kruskal-Wallis test followed by posthoc Benjamini-Hochberg correction adjustment, * p < 0.05, ** p < 0.01, *** p < 0.001


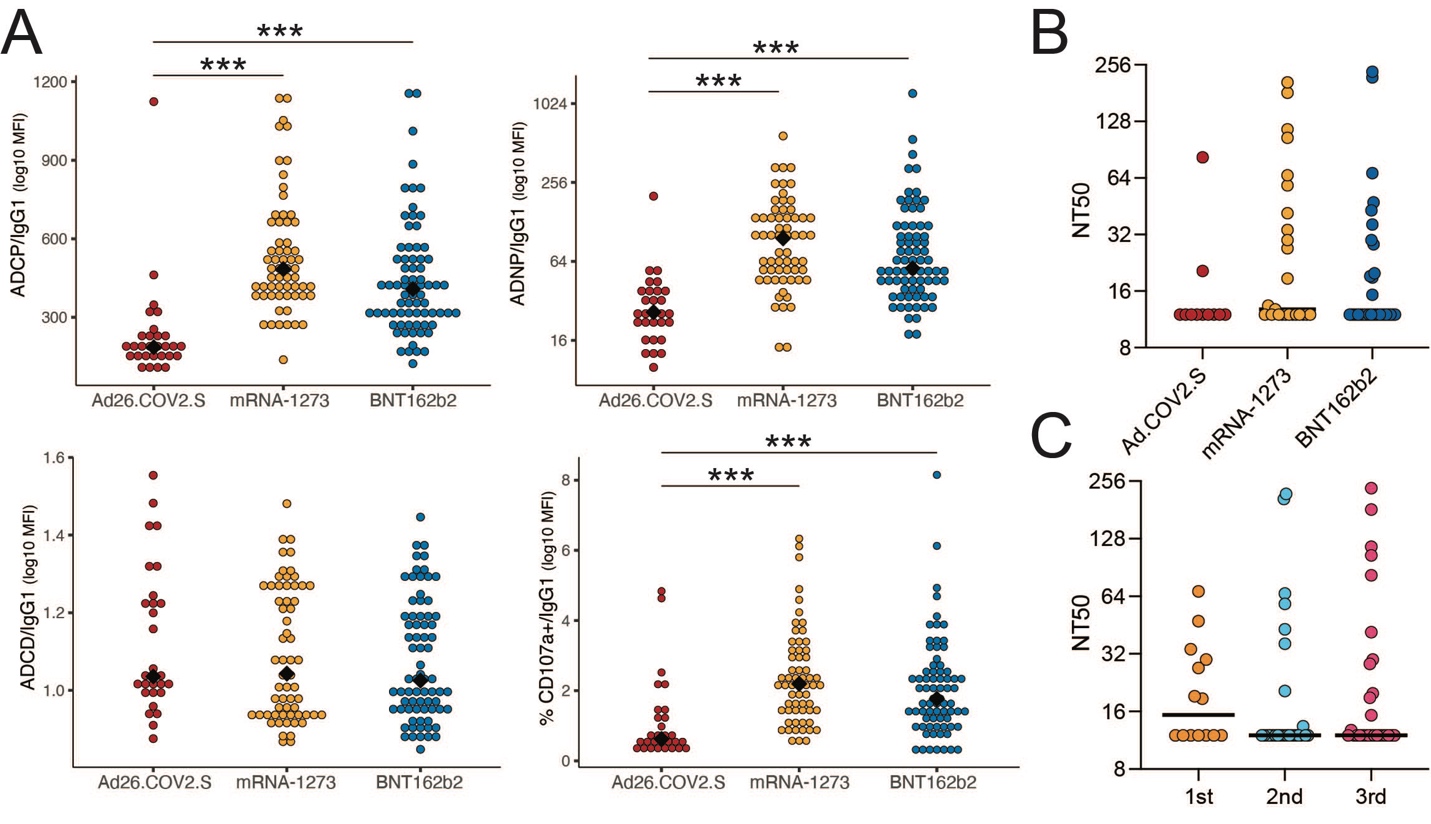


Figure S2. Maternal effector functions and neutralization by vaccine platform
(A) The dot plots show the Spike-specific IgG1 titer-adjusted antibody-dependent cellular phagocytosis (ADCP), antibody-dependent neutrophil phagocytosis (ADNP), antibody-dependent complement deposition (ADCD) and antibody-dependent NK cell degranulation, as measured by % CD107a + NK cells, in maternal samples. Titer adjustment was performed by dividing the functional readout by the log10 IgG1 MFI. Significance was determined by Kruskal-Wallis test, * p < 0.05, ** p < 0.01, *** p < 0.001.

(B) The dot plot shows the 50% neutralization titer (NT50) from an Omicron pseudovirus neutralization assay in maternal samples, by vaccine platform. Significance was determined by Kruskal-Wallis test. There were no significant differences.

(C) The dot plot shows the 50% neutralization titer (NT50) from an Omicron pseudovirus neutralization assay in maternal samples, by trimester of vaccination. Significance was determined by Kruskal-Wallis test. There were no significant differences.

Figure S3. Vaccine-induced antibody titer and function in maternal blood by trimester of vaccination and features enriched by trimester within vaccine type.

1. Spike-specific IgG2, IgG3, IgM and IgA were measured by Luminex. The dot plots show the titer for mothers who received vaccination in the 1^st^ (orange), 2^nd^ (blue), or 3^rd^ (pink) trimester.
2. The dot plots show the Spike-specific antibody-dependent NK cell activation, as measured by % MIP-1b + and % IFN-g+ NK cells, in maternal blood, based on trimester of vaccination. Significance was determined by Kruskal-Wallis test followed by posthoc Benjamini-Hochberg correction adjustment, * p < 0.05, ** p < 0.01, *** p < 0.001.
3. The bar plot shows the LV1 of LASSO-selected features for the PLSDA built in Figure 2E. The color represents the trimester in which the feature was enriched. The network shows the features that are significantly correlated with LASSO-selected features (|r| > 0.8, p < 0.05). Lines connect significantly correlated features.
4. The bar plot shows the LV1 of LASSO-selected features for the PLSDA built in Figure 2F. The color represents the trimester in which the feature was enriched. The network shows the features that are significantly correlated with LASSO-selected features (|r| > 0.8, p < 0.05). Lines connect significantly correlated features.

Figure S4. Vaccine platform results in different vaccine-induced antibody titer and function in cord blood.

1. Spike-specific IgG2 and IgG3 were measured by Luminex. The dot plots show the titer for cords whose mothers received Ad26.COV2.S (red), mRNA-1273 (yellow) or BNT162b2 (blue). MFI: mean fluorescence intensity.
2. The heatmaps show the spearman correlation of antibody features against the Spike of the ancestral virus versus antibody features against the Spikes from variants of concern in maternal plasma, analyzed separately for each vaccine platform. Orange indicates a positive correlation, * p < 0.05, ** p < 0.01, *** p < 0.001, **** p < 0.0001
3. The dot plots show the Spike-specific antibody-dependent NK cell activation, as measured by % MIP-1b + and % IFN-g+ NK cells, in cord blood. Significance was determined by Kruskal-Wallis test followed by posthoc Benjamini-Hochberg correction adjustment, * p < 0.05, ** p < 0.01, *** p < 0.001.

Figure S5. Vaccine-induced antibodies transferred to cord are highly coordinated

(A-C) The network shows the antibody features that were significantly correlated (|r| > 0.8, p < 0.05) with the LASSO-selected features for the mPLSDA built in Figure 4C (A), Figure 4D (B), Figure 4E (C). Lines connect significantly correlated features.
