# Supplementary material for: Maternal immune response and placental antibody transfer after COVID-19 vaccination across trimester and platforms
Source: Nat Commun. 2022 Jun 28;13:3571. doi: 10.1038/s41467-022-31169-8 (PMC9239994; doi:10.1038/s41467-022-31169-8)
Supplement: Supplementary file 5 — Reporting Summary [file 41467_2022_31169_MOESM5_ESM.pdf]

nature portfolio

Corresponding author(s): Edlow, Andrea G.

Last updated by author(s): Apr 26, 2022

Reporting Summary

Nature Portfolio wishes to improve the reproducibility of the work that we publish. This form provides structure for consistency and transparency in reporting. For further information on Nature Portfolio policies, see our Editorial Policies and the Editorial Policy Checklist.

Statistics

For all statistical analyses, confirm that the following items are present in the figure legend, table legend, main text, or Methods section.

n/a	Confirmed	
☐	☒	The exact sample size (*n*) for each experimental group/condition, given as a discrete number and unit of measurement
☐	☒	A statement on whether measurements were taken from distinct samples or whether the same sample was measured repeatedly
☐	☒	The statistical test(s) used AND whether they are one- or two-sided<br>*Only common tests should be described solely by name; describe more complex techniques in the Methods section.*
☐	☒	A description of all covariates tested
☐	☒	A description of any assumptions or corrections, such as tests of normality and adjustment for multiple comparisons
☒	☐	A full description of the statistical parameters including central tendency (e.g. means) or other basic estimates (e.g. regression coefficient) AND variation (e.g. standard deviation) or associated estimates of uncertainty (e.g. confidence intervals)
☐	☒	For null hypothesis testing, the test statistic (e.g. F, t, r) with confidence intervals, effect sizes, degrees of freedom and P value noted<br>*Give P values as exact values whenever suitable.*
☒	☐	For Bayesian analysis, information on the choice of priors and Markov chain Monte Carlo settings
☒	☐	For hierarchical and complex designs, identification of the appropriate level for tests and full reporting of outcomes
☒	☐	Estimates of effect sizes (e.g. Cohen's *d*, Pearson's *r*), indicating how they were calculated

Our web collection on statistics for biologists contains articles on many of the points above.

Software and code

Policy information about availability of computer code

Data collection	No software was used for data collection.
Data analysis	Data analysis was performed in GraphPad Prism version 8.0 or R (version 4.0.0). No custom code was produced. Multivariate analysis was performed using the systemseRology R package v1 (https://github.com/LoosC/systemsseRology)

For manuscripts utilizing custom algorithms or software that are central to the research but not yet described in published literature, software must be made available to editors and reviewers. We strongly encourage code deposition in a community repository (e.g. GitHub). See the Nature Portfolio guidelines for submitting code & software for further information.

Data

Policy information about availability of data

All manuscripts must include a data availability statement. This statement should provide the following information, where applicable:

- Accession codes, unique identifiers, or web links for publicly available datasets
- A description of any restrictions on data availability
- For clinical datasets or third party data, please ensure that the statement adheres to our policy

The Systems Serology and ELISA data generated in this study are provided in Source Data Files 1 and 2.

Field-specific reporting

Please select the one below that is the best fit for your research. If you are not sure, read the appropriate sections before making your selection.

☒ Life sciences ☐ Behavioural & social sciences ☐ Ecological, evolutionary & environmental sciences

For a reference copy of the document with all sections, see nature.com/documents/nr-reporting-summary-flat.pdf

Life sciences study design

All studies must disclose on these points even when the disclosure is negative.

Sample size	Sample size was selected based on sample availability.
Data exclusions	Participants on immunomodulating medications were not included. These criteria were pre-established.
Replication	All experimental assays were done in two independent experiments or with 2 donors. Only highly correlated replicates were used. The average of the replicates was used.
Randomization	There was no randomization. Study groups were balanced for maternal age, gravidity, parity, and pre-pregnancy BMI/obesity.
Blinding	Researchers were blinded when performing experimental assays.

Reporting for specific materials, systems and methods

We require information from authors about some types of materials, experimental systems and methods used in many studies. Here, indicate whether each material, system or method listed is relevant to your study. If you are not sure if a list item applies to your research, read the appropriate section before selecting a response.

Materials & experimental systems

n/a	Involved in the study	
☐	☒	Antibodies
☐	☒	Eukaryotic cell lines
☒	☐	Palaeontology and archaeology
☒	☐	Animals and other organisms
☐	☒	Human research participants
☒	☐	Clinical data
☒	☐	Dual use research of concern

Methods

n/a	Involved in the study	
☒	☐	ChIP-seq
☐	☒	Flow cytometry
☒	☐	MRI-based neuroimaging

Antibodies

Antibodies used	anti-human CD66b Pacblue (Biolegend, Cat 305112, clone G10F5); Anti-guinea pig complement C3 goat IgG fraction, fluorescein-conjugated (MP BioProducts, sku 0855385, cat: 55385, lot 02164); anti-CD107a BV605 (Biolegend, Clone H4A3, cat 328634); anti-CD56 PE-Cy7 (BD Biosciences, clone B159, cat 335791); and anti- CD3 APC-Cy7 (Biolegend, clone (clone UCHT1, cat 300426); BV421 anti-huMIP1beta (BD Biosciences, clone D21-1351, cat 562900); PE anti-huIFNgamma (Biolegend, clone B27, cat 506507); Mouse Anti-Human IgG1 Fc-PE (Southern Biotech, clone HP6001, cat 9054-09); Mouse Anti-Human IgG2 Fc-PE (Southern Biotech, clone HP6002, cat 9070-09); Mouse Anti-Human IgG3 Hinge-PE (Southern Biotech, clone HP6050, cat 9210-09); Mouse Anti-Human IgA1-PE (Southern Biotech, clone B3506B4, cat 9130-09); Mouse Anti-Human IgM-PE (Southern Biotech, clone UHB, cat 9022-09); horseradish peroxidase (HRP)-conjugated goat anti-human IgG antibody (Bethyl Laboratories, Catalog # A80-219P, lot 20).
Validation	Antibodies from Biolegend and BD Biosciences were validated by immunofluorescent staining with flow cytometric analysis. Antibody from Bethyl Laboratories were validated by ELISA. Antibodies from Southern Biotech were validated by ELISA, FLISA, and flow cytometry. Antibodies from MP Bio were validated by: "Antibody titer of the IgG fraction is standardized with an in-house control by immunoelectrophoresis. The IgG fraction is tested for purity and specificity at 40 mg/ml using immunoelectrophoresis. The product is mostly goat IgG; no trace of albumin is detected. This product shows reactivity to guinea pig complement C3; cross-reactivity to other species may exist. Antibody activity to other serum proteins is not present."

Eukaryotic cell lines

Policy information about cell lines

Cell line source(s)	THP-1: ATCC, 293T: ATCC
Authentication	Cell lines were authenticated by ATCC, and ATCC provides a certificate of analysis with all cell lines.

March 2021

Mycoplasma contamination	Performed by ATCC.
Commonly misidentified lines (See ICLAC register)	No commonly misidentified cell lines were used in the study.

Human research participants

Policy information about studies involving human research participants

Population characteristics	Eligible participants were pregnant, greater than or equal to 18 years old, able to provide informed consent, and received the Ad26.COV2.S, mRNA-1273, or BNT162b2 COVID-19 vaccine during pregnancy.
Recruitment	Pregnant individuals at two tertiary care centers were approached for enrollment in the COVID-19 pregnancy biorepository study between January 2021 and September 2021, Protocol #2020P003538, approved by Mass General Brigham Institutional Review Board (IRB). A study questionnaire combined with abstraction of data from the electronic health record (unified across both hospitals) was used to determine history of prior SARS-CoV-2 infection, timing of COVID-19 vaccine doses, and type of COVID-19 vaccine received. While research participation was voluntary, self-selection into a research study would not be expected to impact vaccine responses as assessed by blood draws at standardized intervals before/after vaccination.
Ethics oversight	Protocol #2020P003538, approved by Mass General Brigham Institutional Review Board

Note that full information on the approval of the study protocol must also be provided in the manuscript.

Flow Cytometry

Plots

Confirm that:

☒ The axis labels state the marker and fluorochrome used (e.g. CD4-FITC).

☒ The axis scales are clearly visible. Include numbers along axes only for bottom left plot of group (a 'group' is an analysis of identical markers).

☒ All plots are contour plots with outliers or pseudocolor plots.

☒ A numerical value for number of cells or percentage (with statistics) is provided.

Methodology

Sample preparation	For ADCP, THP-1 cells were obtained from ATCC and passaged in R10+beta mercaptoethanol. For ADNP, leukocytes were isolated from fresh peripheral whole blood using Ammonium-Chloride-Potassium. NK cells were isolated from buffy coats using negative selection and ficoll gradient. All cells were fixed with 4% PFA prior to flow cytometry analysis.
Instrument	iQue (intellicyt)
Software	Flow cytometry data was analyzed in Forecyt (Sartorius)
Cell population abundance	For ADCP (THP-1s), at least 2000 cell events per well. For ADNP (Neutrophils), at least 2000 cells per well.<br>For NK cells, at least 1000 cells/well
Gating strategy	For ADNP, Neutrophils were defined as CD66b+ (Pacblue+) leukocytes. Neutrophils that had phagocytosed beads were defined as FITC+. For ADCP, THP-1s that had phagocytosed beads were defined as FITC+. For both ADCP and ADNP, FITC+ gates were defined based on negative and positive assay controls. For ADNKD, NK cells were defined as CD56+/CD3-. Activity was defined as %CD107a+, %IFNg+, %MIP-1b+. Gates were defined based on negative and positive assay controls.

☒ Tick this box to confirm that a figure exemplifying the gating strategy is provided in the Supplementary Information.
